# Remote court hearing as a judicial response to the COVID-19 outbreak: An impact assessment and suggestions for improvement

**DOI:** 10.7189/jogh.11.03051

**Published:** 2021-03-27

**Authors:** Xingmei Zhang

**Affiliations:** School of Law Jilin University, Changchun, Jilin, China

The COVID-19 outbreak caused almost all countries to declare public health emergencies. The government, medical institutions and even civil societies are taking up measures to deal with the pandemic [1]. Despite the presence of a health emergency, litigation must continue. Previous studies have shown that non-pharmaceutical interventions such as lockdowns, social distancing and school closures play an important role in reducing virus transmission [2]. In that sense, litigation may continue but without face-to-face contact. As a response, the judiciary has come up with remote court hearings. Although different countries and regions have mixed feelings on remote court hearings, the pandemic has necessitated its application. Evidently, even the conservative US Supreme Court has begun to try to use teleconferences to hold court hearings remotely, both for health and safety reasons [3].

China has a relatively positive attitude towards remote court hearings compared to other countries. The pandemic has accelerated the development and application of remote court hearing in China. This is evident as cases handled through electronic litigation have increased considerably [4,5]. However, the application of remote court hearings to observe social distancing may also create challenges for the rules during court hearings. In order to improve the validity of remote court hearings as a response to public health emergencies and to standardise its application, it is necessary to assess its impact.

China’s remote court hearing system can give researchers an insight into this system and be applied in their own countries. As it currently seems, remote court hearing is here to stay, even after the pandemic. As such, we need to develop strategies and techniques to improve remote court hearing by asking ‘Is it necessary to develop remote court hearing, and does it fit in with the public health emergencies? How do we evaluate the impact of remote court hearing, and how do we standardise this system to enhance the ability of judiciary to deal with public health emergencies and promote the modernisation of judicature?’

## THE APPLICATION OF REMOTE COURT HEARING IN CHINA

I analyzed the judgement documents from China Judgements Online[6]. The last retrieval date is December 16, 2020, which include only mainland China. The key words used in the judgement documents retrieval are Remote, WeChat, QQ, Video, Online, Internet, Network, Electronic, Cloud, Platform and Micro. A 2nd retrieval of the Trial Process was conducted, after which manual screening was performed. I excluded the judgements made by the Internet Court as a special court, those merely by the witnesses or appraisers in a remote court hearing, and repeated and irrelevant judgements. A total of 7221 judgement documents which demonstrably use remote court hearings were obtained. From these documents, the annual distribution, regional distribution, procedural distribution and other relevant data were obtained. The data collected reflects the impact of remote court hearing in China, especially during the pandemic.

It can be see that the COVID-19 outbreak has had a significant impact on the application of remote court hearings. The judiciary regards remote court hearing as an important measure to deal with the current outbreak.

**Figure 1:**
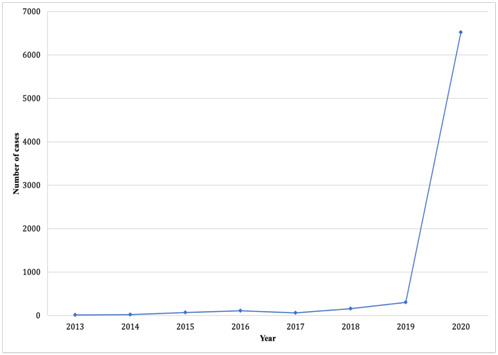
Annual distribution of remote court hearing in mainland China.

As shown in **Figure 1**, there were 6 cases that were tried through remote court hearing in 2013, 18 cases in 2014, 68 in 2015, 102 in 2016, 55 in 2017, 154 in 2018, 298 cases in 2019 and 6520 cases in 2020. The number of cases tried through remote court hearings in 2020 significantly increased, with 350 judgement documents stating that the application of remote court hearing was entirely due to COVID-19.

Based on **Table 1**, the application of remote court hearing was implemented mainly in Zhejiang from 2013 to 2016. By the year 2019, remote court hearing has been practiced in 31 provinces, autonomous regions and municipalities in mainland China. According to other data, as of 2019, 58.2% of China’s courts support remote court hearings [7]. In 2020, the application rate of remote court hearing in these areas significantly increased. According to China’s relevant norms, the pilot courts that can hold remote video court hearing include Intermediate People’s Courts and Local People’s Courts under the jurisdiction of Beijing, Shanghai, Nanjing, Suzhou, Hangzhou, Ningbo, Hefei, Fuzhou, Xiamen, Jinan, Zhengzhou, Luoyang, Wuhan, Guangzhou, Shenzhen, Chengdu, Guiyang, Kunming, Xi’an and Yinchuan, Intellectual Property Courts of Beijing, Shanghai and Guangzhou, Shanghai Financial Court and Internet Courts of Beijing, Hangzhou and Guangzhou. However, other areas where there are no pilot courts such as Gansu, Jilin, Liaoning and Heilongjiang have applied remote court hearing, as shown in **Table 1**. Higher People’s Courts in some non-pilot areas have also issued opinions on strengthening and regulating online litigation during the COVID-19 pandemic.

**Table 1:** Regional distribution of remote court hearing in mainland China

Rank	Region	2013	2014	2015	2016	2017	2018	2019	2020	Total
**1**	**Zhejiang**	4	11	40	69	23	26	56	1089	1318
**2**	**Beijing**	0	0	0	0	3	4	71	1127	1205
**3**	**Hebei**	0	0	0	1	2	1	1	672	677
**4**	**Shandong**	0	0	2	0	0	0	2	472	476
**5**	**Henan**	0	0	0	1	3	7	2	430	443
**6**	**Liaoning**	0	2	1	2	1	6	9	280	301
**7**	**Shanghai**	0	0	0	0	0	9	2	249	260
**8**	**Sichuan**	0	0	1	5	3	9	18	213	249
**9**	**Jilin**	0	0	2	4	2	5	23	192	228
**10**	**Jiangsu**	0	0	4	8	0	0	1	181	194
**11**	**Yunnan**	0	0	1	0	2	6	5	160	174
**12**	**Guangdong**	0	0	0	0	0	2	2	169	173
**13**	**Guizhou**	0	0	1	3	3	30	41	93	171
**14**	**Heilongjiang**	0	1	0	0	2	6	9	151	169
**15**	**Chongqing**	0	0	8	3	2	2	1	136	152
**16**	**Fujian**	0	0	0	0	0	1	1	137	139
**17**	**Hunan**	0	0	1	0	1	1	2	124	129
**18**	**Jiangxi**	0	0	3	0	2	2	4	114	125
**19**	**Xinjiang**	0	0	0	0	0	0	1	87	88
**20**	**Hubei**	1	0	3	1	0	9	9	63	86
**21**	**Tianjin**	0	0	0	0	0	0	1	82	83
**22**	**Guangxi**	0	2	0	0	0	4	1	73	80
**23**	**Anhui**	0	0	0	1	0	2	1	73	77
**24**	**Gansu**	1	0	1	2	0	8	13	51	76
**25**	**Shaanxi**	0	1	0	0	1	5	11	25	43
**26**	**Inner Mongolia**	0	0	0	1	0	4	1	24	30
**27**	**Qinghai**	0	0	0	0	1	0	2	23	26
**28**	**Shanxi**	0	1	0	0	1	2	3	11	18
**29**	**Tibet**	0	0	0	0	2	3	3	8	16
**30**	**Ningxia**	0	0	0	1	0	0	2	10	13
**31**	**Hainan**	0	0	0	0	1	0	0	1	2
	**Total**	6	18	68	102	55	154	298	6520	7221

**Figure 2:**
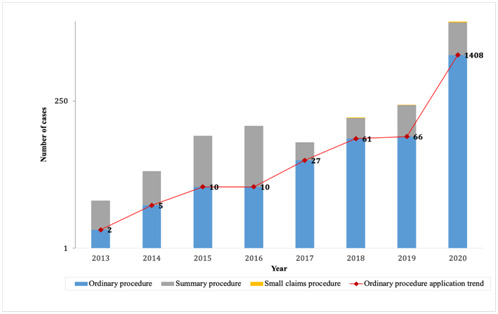
Procedural distribution chart for remote court hearing in mainland China.

From the 7221 cases that demonstrably use remote court hearings, 5525 cases of remote court hearings were for first instance procedures; 1640 cases were for second instance procedures; and 56 cases were for retrial procedures. **Figure 2** shows that from the 5525 first instance cases done through remote court hearings, 1589 cases (including 2 cases in 2013, 10 cases in 2014, 10 cases in 2015, 10 cases in 2016, 27 cases in 2017, 61 cases in 2018, 66 cases in 2019 and 1408 cases in 2020) were of ordinary procedures, and 3734 cases (including 4 cases in 2013, 13 cases in 2014, 58 cases in 2015, 89 cases in 2016, 26 cases in 2017, 71 cases in 2018, 148 cases in 2019 and 3325 cases in 2020) were of summary procedures. Small claims procedure was applied in 202 cases (including 3 cases in 2018, 3 cases in 2019 and 196 cases in 2020). It can be seen from **Figure 2** that the cases applicable to remote court hearing by ordinary procedures have been increasing. This means that remote court hearing is no longer limited to the hearing of simple cases. It is now widely used by the court, and has gradually become the norm for court hearings.

The practice of remote court hearing in China reflects the demand to innovate during public health emergencies. In 2020, the number of cases, geographical scope and procedural scope of remote court hearings all showed a rapid growth trend which allowed the process of litigation to continue despite the public health emergency. However, the existing practices of remote court hearing in China present a great variety of forms, which means that remote court hearings urgently need theoretical care and normative guidance.

Regarding the scope of procedures, both the summary procedures and small claims procedures, as well as ordinary procedures, can be tried through remote court hearings. In addition, not only cases of first instance procedures can be conducted through remote court hearings, but also cases of second instance procedures or retrial procedures are under the category of remote court hearings. In a geographical scope, remote court hearings are not only limited to the pilot courts. There are variations on how remote court hearings are conducted. Some courts rely on the platforms that they themselves have developed (eg, COFCO Group Co., Ltd v. Jinan Pujia Wine Industry Co., Ltd and Alibaba Advertising Co., Ltd, the executive judge tried the case through the Online Trial Platform of Zhejiang Court). Others use third-party platforms such as QQ (eg, Wu Xuejian v. Xia Zonggang, the defendant participated in the hearing through QQ) and WeChat (eg, Chen v. Wei, the defendant Wei participated in the hearing through WeChat). Some courts adopt video calls (eg, Wang Guihong v. Zhao Shihe and Zhao Zhongqing, the plaintiff participated in the court hearing through online video), and some use voice calls (eg, Bai Ruixiang v. Qi Jianguang, the defendant participated in the hearing through telephone). Others start with the request or consent of only one party (eg, Huang v. Gao, the court allowed the plaintiff to participate in the hearing in the form of network video), while some commence their hearings with the requests or consent of both parties (eg, Li v. Yu, with the application and consent of both parties of the case, the court allowed the use of audio-visual transmission technology for court hearing).

Court hearings are concentrated in the fields of litigation, legal communication and realisation of one’s rights. Whether its construction is normative or not is directly related to the judicial power and the rights of the parties in the case. Even if remote court hearings are necessitated by public health emergencies, justice, parties’ rights and other factors of litigation must be carefully considered and observed. Therefore, it is necessary to assess the impact of remote court hearings and develop appropriate responses and strategies.

## NECESSARY EXISTENCE OF REMOTE COURT HEARING

Remote court hearing, also called online court hearing, refers to the online process of hearing proceedings without the physical presence of the subjects of litigation using computer and internet as a medium to transmit texts, voices, images and related information. Compared with the traditional way of court hearing, remote court hearing is technology dependent, relies on network stability, flexible and paperless. The characteristics of remote court hearing can also improve the court’s ability to deal with public health emergencies.

Remote court hearings help reduce the risk of infection and improve their court appearance rate. In an age centred on information and internet technology, online communication slowly removes barriers between people, which makes us connect more easily to others. Remote court hearings enable both parties to communicate freely, and makes hearings more convenient to all parties involved. As long as the litigants have the equipment and network, they can enter the court hearing in a non-face-to-face manner instead of attending hearings in person. Remote court hearings reduce contact between people and uses technology to transmit communication information and complete online statements, defences, evidence presenting, cross-examinations and inquiries. In the application of remote court hearing, litigation documents and evidence materials are presented, transmitted, reviewed and kept on file electronically. This helps to further reduce contact transmission.

In summary, remote court hearings are borne from a need to continue litigation despite public health emergencies. As scholars have said, modern justice must be effectively accessible to all, not just theoretically [8]. Even during the COVID-19 outbreak, the right of the litigants to access to justice should also be guaranteed. Remote court hearing may offer the most promising way of increasing access to justice around the world [9]. In this sense, we should affirm the necessity of remote court hearing and observe its development.

## LEGITIMACY OF REMOTE COURT HEARING

Aristotle says that the precision of things needs to be found in the nature of the subject [10]. The nature of remote court hearing is court hearing procedure. As a novel method, it is necessary to scrutinise and analyse remote court hearing legitimacy by referring to the factors of court hearing.

According to the theory of litigation, court hearing is an activity of directness, orality, ceremony and transparency [11]. These factors bear the substantive justice value of litigation. Emphasising these factors can make judges better determine the facts of the case, form them inner confidence and highlight the authority of the judgement. Remote court hearing is the transformation from face-to-face hearing to non-face-to-face hearing, formerly conducted from a judicial theatre and currently to a judicial square [12,13]. Although the transformation and the development make it possible for disputes to be settled in a non-contact and convenient way, remote court hearing may also impact the factors of court hearing in different degrees.

**Figure:**
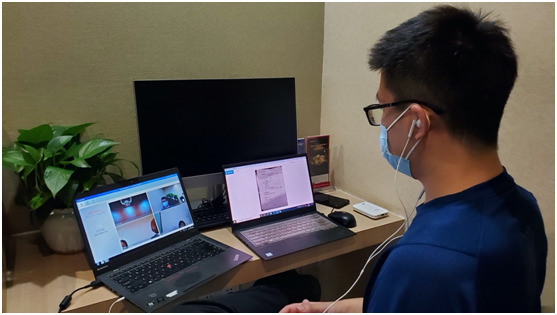
Photo: A lawyer is preparing for a remote court hearing on September 11, 2020 (from Kaiwen Li, used with permission).

First, the evidence the judge sees at the court is what has been processed electronically. This sort of evidence is not equivalent to the original evidence. Thus, remote court hearing reduces the possibility of the judge having direct access to the original evidence. Second, due to non-contact of the subjects, the possibility for the judge to grasp the details and particulars of the court hearing decreases when they observe the voice, intonation, manner, expression and movement of the parties. In a remote court hearing without matching simultaneous HD video technology, judges’ hearing and observation could be hindered. However, direct observation, plus the knowledge of psychology and physiology to find the truth of the case, is the essence of direct hearing. Third, technical media, like a barrier, lowers both the psychological deterrent of the parties and the binding force of the court hearing. Accordingly, the probability of arbitrariness and falsity of the statements made by the parties in the hearing process increases. This especially true when the parties participate in the remote court hearing from their homes, as the casual living environment and the stately atmosphere of a court hearing constitute a striking contrast between venues. Casual dresses, lazy sitting postures, frequent exits, and other improper behaviours are likely to occur [14]. Fourth, the multi-dimensional hearing venues at different locations increase the difficulty for the public to watch the hearing. How to protect the rights of the public for watching the remote court hearing and how to regulate the behaviour of the public in watching the remote court hearing deserve our concern. Last but not least, remote court hearing may lead to excessive transparency. Participants in the case might record the voices, scenes, and screenshots, thus touching on the boundary of judicial transparency, challenging people’s private interest.

Remote court hearing has an impact of different degrees on the factors such as directness, orality, ceremony and transparency of the court hearing. The impact more likely runs counter to the fair value of the elements of the hearing. This means that although it is necessary to conduct remote court hearings during public health emergencies, the application of this system cannot be generalised.

## FEASIBILITY OF REMOTE COURT HEARING

Only a litigation procedure is the source of legitimacy for a judgement or settlement [15]. Even when dealing with emergencies, court hearing procedures cannot be ignored. In this way, the judicial use of remote court hearing as a response to the public health emergencies is valid. In view of the limitations of remote court hearing, the feasibility of remote court hearing can be considered from the following aspects:

First, in terms of technical operation, the court should be the builder and manager of the remote court hearing platform. By using public power to construct and manage the remote court hearing platform, the court is in a position to support the neutrality and security of the platform and enhance the trust of litigants. Second, remote court hearing should be conducted by video, and the application of video can ensure the clarity, synchronisation and real-time performance of the scenes. Third, in principle, remote court hearing should be a connection between court and court, that is, the subjects applying for remote court hearing should go to the court closest to them for hearing. The public, at different locations, may choose to go to the nearest court to exercise their right to sit in on a hearing. The court may also consider setting up special remote courts in its area for case parties to use. Of course, during public health emergencies, the parties will be required to restrict going outdoors as much as possible. When the litigants must participate in a remote court hearing at home, the judge should remind the parties of court hearing rules and inform them of the legal consequences of misconduct.

In terms of start-up mode, the application of remote court hearing should focus on the parties, attention must be given to the principal position of the parties, and respect must be observed to the procedural options of the parties. Considering the equality of the parties, the start of remote court hearing should be based on the mutual choice of both parties. The court is not completely passive regarding initiation of remote court hearing. Instead, it gives proper play to its command power in terms of the hearing on the premise of the choice made by both parties. The court weighs the situation and determines what is a more appropriate court hearing mode based on the case’s dispute, evidence and characteristics of the procedure.

In terms of scope of application, the scope where remote court hearing is applied expands with careful consideration from simple cases to those of average difficulty, which is a trend in line with the characteristics of the time and the practical needs of litigation societies. What kinds of cases are more suitable for remote court hearings still needs some practical observation and accumulation of cases. However, it is wrong to think that the application of remote court hearing has no rules to follow, and the following two factors deserve attention: In one sense, remote court hearings are applicable to cases where the authenticity of evidence is not controversial. Because remote court hearings impact on the form of existence of traditional evidence and increases the risk of losing authenticity of evidence, if both parties have doubts about the authenticity of the evidence, the application of remote court hearing undoubtedly aggravates their disputes. In another sense, remote court hearings are more suitable for legal trial cases in which direct contact between the subjects of proceedings is not necessary. As a whole, the trial grade from low to high is a trend of weakening the fact trial and strengthening the legal trial. Moreover, with higher trial grade, the phenomenon that the parties’ cross-region attendance on the hearing occasion is more common. Therefore, remote court hearings have more application space in the appeal trial cases or retrial cases.

## CONCLUSIONS

The COVID-19 outbreak has accelerated the application of remote court hearing. Remote court hearing results in an orientation of higher technology and flexibility with less paperwork and contact. These characteristics of remote court hearing help the litigant to access to justice, enhance the litigant procedure participation, and promote the litigation convenience, However, remote court hearing does not always bring about progress, and it also challenges court hearing factors such as the directness, orality, rituals and transparency of the court hearing. Therefore, the application of remote court hearing should not be generalised. By guiding the construction of remote court hearing platforms and technical operations, start-up mode and application scope, it will further strengthen the progress of remote court hearing, weaken its limitations and improve the validity of remote court hearing during public health emergencies.
